# Nutritional education on health beliefs, metabolic profiles, and quality of life among high-risk pregnant women for gestational diabetes mellitus: a randomized controlled trial

**DOI:** 10.1038/s41598-024-78447-7

**Published:** 2024-11-12

**Authors:** Robab Sharifat, Fatemeh Borazjani, Marzieh Araban, Amir H. Pakpour, Kambiz Ahmadi Angali, Saleh Aiiashi

**Affiliations:** 1 Nutrition and Metabolic Diseases Research Center, Clinical Sciences Research Institute, Ahvaz Jundishapur University of Medical Sciences, Ahvaz, Iran; 2 Menopause Andropause Research Center, Ahvaz Jundishapur University of Medical Sciences, Ahvaz, Iran; 3 Department of Nursing, School of Health and Welfare, Jönköping University, Jönköping, Sweden; 4 Social Determinants of Health Research Center, Ahvaz Jundishapur University of Medical Sciences, Ahvaz, Iran; 5 Department of Statistics and Epidemiology, School of Health Sciences, Ahvaz Jundishapur University of Medical Sciences, Ahvaz, Iran; 6 Abadan University of Medical Sciences, Abadan, Iran

**Keywords:** Gestational diabetes mellitus, Health Belief model, Nutrition Education, Nutrition knowledge, Glycemic Index, Endocrinology, Health care

## Abstract

In recent years, nutrition has become increasingly important in treating and managing gestational diabetes mellitus. The Health Belief Model (HBM) is a conceptual framework in health behavior research used in some medical research. The present study aimed to evaluate the effect of glycemic index training based on the HBM on metabolic indicators and pregnant women’s health-related quality of life. In this open-label, parallel-controlled randomized trial, 90 pregnant women from primary health centers in Iran were recruited and randomly assigned to either the intervention group or the control group, using a block randomization method with a block size of six. The intervention group underwent 12 weeks of nutritional education on the glycemic index and load of foodstuff. The control group only received routine health care. Valid and reliable questionnaires included demographic and prenatal information, physical activity, three days of food records, quality of life (SF-12), and HBM constructs. Fasting blood samples were taken at baseline and end of the study, to assess fasting blood glucose (FBS), fasting insulin, hs-CRP, and lipids. Eighty-four pregnant women with an age mean of 30.12 ± 4.35 completed 12 weeks of intervention. At baseline, there were no significant differences between groups in the HBM subscales (*P* > 0.05). However, within the intervention group, there was an increase in perceived susceptibilities (mean change 1.45 ± 4.21; *P* = 0.03) and cue to action (mean change: 1.22 ± 3.38; *P* = 0.02). In the adjusted model, the General Health subscale of Quality of Life showed a significant increase in the comparison between groups (mean change 13.69 ± 29.83 vs. 0.00 ± 29.58; *P* = 0.04). Additionally, the adjusted model revealed a notable difference between the groups in serum hs-CRP level, (mean change -0.877 ± 3.47 vs. -0.067 ± 3.40; *P* = 0.01) and triglyceride level (mean change − 55.24 ± 111.21 vs. 40.92 ± 142.01; *P* = 0.001). However, in the adjusted model, the total cholesterol levels increased between groups (mean change 22.75 ± 66.17 vs. 30.12 ± 61.33; *P* = 0.01) at the end of the study. There was no significant difference in glycemic indices (*P* > 0.05). Participation in a nutrition education program might positively impact nutritional beliefs, behaviors, and some biochemical indicators among pregnant women. Future studies with larger sample sizes and longer follow-ups are warranted.

Trial registration number: IRCTID: IRCT20190227042858N1. Registration date: 2019-07-18.

**Supplementary Information:**

The online version contains supplementary material available at 10.1038/s41598-024-78447-7.

## Introduction

WHO stated” Access to diabetes education is the focus of the second year of the World Diabetes Day 2021-23 theme”^1^. Hence, due to increased sedentary behavior and growing obesity prevalence, women have been more vulnerable to a variety of pregnancy problems in recent decades^2^. Recently, there has been a significant increase in the prevalence of gestational diabetes mellitus (GDM) among pregnant women. In 2022, the pooled global standardized prevalence was 14.0%^3^, while in Iran, the prevalence rate was 7.6% in 2023^4^. Various risk factors such as genetic predisposition, age, weight, environmental pollutants, and diet have been identified^5^. gestational diabetes mellitus doesn’t disappear after childbirth and can develop into type 2 diabetes (T2DM) without changes in diet and lifestyle^6^. Maternal obesity is a significant risk factor, and the prevalence of gestational diabetes mellitus increases with higher pre-pregnancy BMI^7^. A recent meta-analysis showed Pregnant women can benefit from preventive strategies such as diet, exercise, a combination of diet and exercise, and oral hypoglycemic agents can lower the risk of gestational diabetes mellitus compared to control groups^8^. Lifestyle changes like a healthy diet and exercise can also help to prevent gestational diabetes mellitus^9,10^. Pregnancy alters the inflammatory profile and has a diabetogenic effect on metabolism compared to the non-pregnant state^11^.

Dietary control aims to maintain blood glucose levels within the normal range while avoiding hypoglycemia or ketosis due to excessive carbohydrate reduction^12^. The type of carbohydrate consumed also affects serum glucose concentration in diabetes patients^13^.

The Glycemic Index (GI) measures how quickly carbohydrate sources raise blood glucose. Foods are given a score from 0 to 100 based on this ability, with a higher score indicating a quicker blood glucose spike. Foods with a GI value of < 55 are considered low-GI foods^14^. This diet can help control maternal blood glucose, reduce the risk of excessive weight gain during pregnancy, and lower the risk of birth defects^15^. Research has shown that consuming low-GI foods is associated with lower levels of C-reactive protein, blood glucose, glycosylated hemoglobin, and insulin^16^.

Nutritional education, such as teaching pregnant women about a low glycemic index (LGI) diet, can help control blood glucose and prevent health issues. Health education utilizing theories or models is crucial for promoting healthy behaviors in this population^17^. The Health Belief Model (HBM) is a key strategy for improving health status, as it emphasizes the importance of perceived susceptibility, severity, benefits of behavioral change, and perceived barriers^18^. Implementing the HBM can positively impact lifestyle changes and the management of chronic disease symptoms^19^. Moreover, the (HBM) model is gaining recognition as a more effective framework for nutrition education than traditional approaches due to its emphasis on individuals’ perceptions of health risks and advantages, which boosts engagement and motivation. By tackling psychological elements such as perceived susceptibility, severity, benefits, and obstacles, HBM shapes dietary habits more thoroughly than standard methods. Furthermore, HBM-focused interventions aim to enhance self-efficacy, pinpoint personal challenges to healthy eating, and cultivate intrinsic motivation for changing behaviors^18^. Consequently, research indicates that programs based on HBM result in significantly better improvements in dietary knowledge, attitudes, and behaviors^20,21^.

Considering the structure of the HBM and its components seems appropriate for educational intervention. Therefore, the present study is designed to evaluate the effect of glycemic index training based on the HBM model on metabolic profile and health-related quality of life among pregnant mothers at risk of gestational diabetes mellitus referring to primary health centers.

## Methods

### Study population

The current study is an open-label, parallel-controlled randomized trial that was conducted among pregnant women referred to the primary health centers, in Omidiyeh, southwest of Iran, from September 2020 to February 2021.

This study included pregnant women with a gestational age between 12 and 16 weeks who were literate, had a single pregnancy, and had at least one risk factor for gestational diabetes mellitus. These risk factors included: being aged 25 or older^22,23^, having a family history of type 2 diabetes in first-degree relatives, having a pre-pregnancy BMI over 25 if overweight or over 30 if obese, a history of gestational diabetes mellitus or glucose intolerance, having a previous pregnancy with a baby weighing over 4000 g, experiencing a premature baby or miscarriage^24,25^ during a previous pregnancy. Pregnant women were excluded from the study if they had type 2 diabetes, thyroid disease, cardiovascular disease, respiratory disease, a history of taking medications that affect blood glucose levels (such as corticosteroids), or if they were not actively attending educational sessions.

The entire process of study was approved by the Ethics Committee of Ahvaz Jundishapur University of Medical Sciences (Ethical NO. IR.AJUMS.REC.1397.600) and registered with the Iranian Clinical Trials Registry (IRCT registration number: IRCTID: IRCT20190227042858N1).

### Participant recruitment and screening

The total sample size was calculated based on the result of FBS (mg/dl) reported in the previous study^26^ among participants with gestational diabetes mellitus in the intervention group who received a DASH eating plan that has the same characteristics as a low-glycemic index diet. considering a mean (SD) of 84.81 (8.16), power of 90%, 95% confidence, and accounting for 30% dropout, a calculated sample size of 45 women in each group. All participants signed written informed consent. The present study was designed based on the CONSORT statement for randomized clinical trials^27^.





**Fig. 1:**
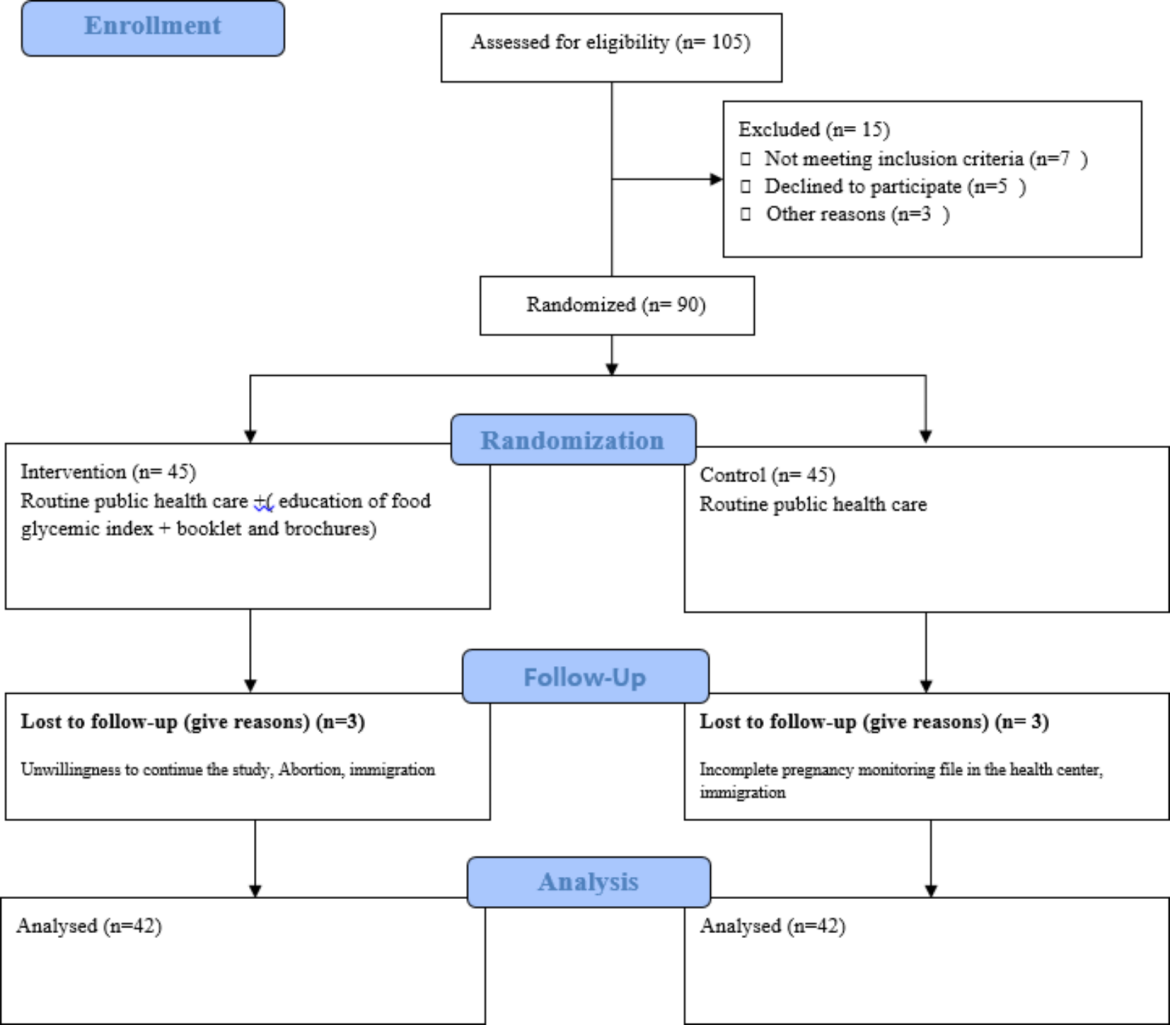
CONSORT flow diagram.

One Hundred and Five pregnant mothers were initially evaluated, and ninety were eligible to enter the study. Fifteen pregnant mothers were not included in the study due to not meeting the inclusion criteria, declining to participate, and other reasons. Ultimately, ninety pregnant mothers were randomly assigned to two groups using a block randomization procedure. Each group consisted of Forty-five mothers, with a block size of six, ensuring equal proportions. The random number list was generated by a computer and executed by an individual not involved in the study. It is important to note that, due to the education-based nature of the study, the researcher was not blinded. However, both the data analyst and laboratory analyst were blinded to the study groups. Eligible participants were informed about the study’s aims and provided signed written informed consent before entering the study. Figure 1 illustrates the CONSORT flow chart of the participant’s enrollment.

### Study design and interventions

At the baseline, expert researchers completed socio-demographic and health-related questions, HBM, SF-12 questionnaires, and a short version of the International Physical Activity Questionnaire (IPAQ) for all participants in both groups. After that, the educational intervention was held in 2 face-to-face sessions for each group of 8–10 people for 90 –60 min in primary health centers and then continued by sending messages every other day through WhatsApp group/text message. The contents of the booklets and brochures were prepared based on the latest guidelines^28,29^ and nutritional references^30^, and the foods were classified according to the dietary glycemic index and load, providing more accurate information about the impact of a food on postprandial glycemia. The GI value of each food item was obtained from the international table and the available lists of Iranian foods^31,32^. In the first session, participants received training on food, nutrition, physiological changes, and eating habits during pregnancy. An educational intervention was conducted based on the Health Belief Model theory. Perceived susceptibility of pregnant women to developing gestational diabetes mellitus and experiencing improper weight gain is increased by the consumption of calorie-dense, nutrient-less foods high in sugar, such as sweets, sugar-sweetened beverages, starchy vegetables, refined grains, and fat, such as fast foods. The Perceived severity is described as follows: if they are affected with gestational diabetes mellitus, they are at risk for stillbirth, macrosomia, and cesarean section. Perceived susceptibility and severity were taught through lectures and by showing relevant pictures as well.

Barriers to appropriate dietary practices include difficulty accessing vegetables, fruits, and whole grains, lack of time for cleaning vegetables, insufficient information about pregnancy care, and unfamiliarity with proper cooking methods were discussed and solutions to the barriers were explained. The benefit of preventing gestational diabetes mellitus is important for both maternal and fetal health. The group discussion covered both barriers and benefits. Self-efficacy to follow the appropriate dietary practices was raised by inviting pregnant women as a role model for consuming a balanced diet and normal weight.

Advice from a health care professional was used as a Cue to action to adhere to self-care practice for pregnant women. This advice was as follows: avoid sugary and oily food items. Seek support from family and obtain information from health centers and media sources. Knowledge of incorporating fresh fruits, vegetables, nuts, and low-fat dairy as an appropriate snack while skipping meals can unfavorably impact blood glucose was also discussed. Subsequently, brochures and booklets with the same content were also distributed among the participants.

In the second session, we discussed the educational content on how the glycemic index and glycemic load of food items affect maternal blood glucose, gestational diabetes mellitus prevalence, and complications for both mother and fetus. Talking about the risk of gestational diabetes mellitus linked to high glycemic index cereals (such as white rice, white bread, and pasta) as well as starchy vegetables (including potatoes and other starchy vegetables). The expression of the GI value of rice is mainly influenced by the cooking method, cooking time, and the amount of added liquid and other added ingredients that will affect the postprandial glycemic response. During the educational sessions, lectures, and group discussions were conducted on two specific topics: “healthy diet” and “healthy lifestyle**”.** Hence, it is advisable to stay active and take essential nutrients like iron, multivitamins, minerals, and folic acid. Consuming meals at regular intervals earlier in the day contributes to better blood glucose control during pregnancy.

Participants in the control group received their routine public health education. At the end of the intervention follow-up period, they also received educational materials. After 12 weeks of intervention, all study questionnaires were completed again by participants in both groups.

## Outcome measures

### Primary outcome

The primary outcome involves assessing serum levels of fasting blood glucose (measured in mg/dL), results from the Oral Glucose Tolerance Test (OGTT, in mg/dL), high-sensitivity C-reactive protein (hs-CRP, in mg/mL), fasting insulin levels (in µU/mL), and differences in Health Belief Model scores between the intervention and control groups.

### Secondary outcomes

The secondary outcomes include an evaluation of the average differences in dietary intake, physical activity, gestational weight gain (GWG, in kg), health-related quality of life, as well as insulin sensitivity and resistance measured by the Homeostasis Model Assessment (HOMA-IS and HOMA-IR). Additionally, we will assess serum lipid profiles (measured in mg/dL) in both intervention and control groups.

## Study tools

### Assessment of the health belief model questionnaire

The HBM questionnaire has a researcher-made structure and consists of 53 items divided into six categories derived from existing healthcare literature on food glycemic levels, pregnant blood glucose levels, and the health condition of the fetus (supplement Table 1). These subsections included: (1) Perceived susceptibility consisted 6 questions to evaluate; weight changes and blood glucose concentration and the impact of food groups on pregnant women’s health condition, (2) Perceived severity which consisted of 9 questions to examine the negative effects of higher simple carbohydrate consumption on mother health and fetus growth, (3) Perceived barriers were used to recognize the barriers preventing pregnant women from attending regular check-ups and accessing a healthy diet, utilizing an 8-item reverse-rating system, (4) Perceived benefit which included six items that measure the benefits of low glycemic index food items. (5) Cue to action included four questions in terms of pregnant women’s perspective on the availability and benefits of high-fiber cereal and vegetables for the prevention of gestational diabetes mellitus, and (6) Self-efficacy which consisted of six items to evaluate the individual’s ability to manage gestational blood glucose by healthy foods consumption. (7) Also, 8 items were used for evaluation of pregnant women’s knowledge about the food choices with lower glycemic index and the effect of cooking type on the pregnant women’s glycemic status. For all items, responses were rated from 1 (strongly disagree) to 5 (strongly agree), except for the perceived barriers, and 3 items of perceived susceptibility which scored in a reverse manner.

**Table 1:** Baseline characteristics of the study population.

characteristics	Intervention *N* = 42	Control *N* = 42	P1-value between groups
Maternal age(year)	29.97(5.86)	30.84(5.67)	0.4
Pre-BMI (kg/m^2^)	26.40(4.67)	27.93(5.34)	0.1
2nd trimester weight gain(kg)	6.27(4.85)	6.23(3.53)	0.9
MET (min/week)			
Baseline	979(1022.1)	508.5(837.1)	0.01
Follow-up	896(1047)	578.7(1087.74)	0.1
P-value	0.7	0.8	
Income status (%)			
Weak	20.5	11.4	0.5
Moderate	38.6	43.2	
Good	40.9	45.5	
Living with the old person (%)			
Yes	15.9	15.9	1
No	84.1	84.1	
Number of children (%)			
Not	2.3	27.3	0.004
Only one	29.2	25.0	
> One	68.2	47.7	
Mother job status (%)			
Employed	6.8	6.8	1
Housewife	93.2	93.2	
Pregnancy intention (%)			
Planned	92.9	85	0.2
Unplanned	7.1	15	
Mother education status (%)			
< 12year	69	72.7	0.7
> 12year	31	27.3	
Spouse education status (%)			
< 12year	68.3	72.1	0.7
> 12year	31.7	27.9	
Folic acid supplement (%)			
Regular use	100	100	1
Irregular use	0	0	
Iron supplement (%)			
Regular use	100	100	1
Irregular use	0	0	

Data presented as mean (SD) or %.

The validity of the HBM questions was evaluated by the content validity index (CVI) and content validity ratio (CVR). The CVI and CVR for the health belief model questionnaire were 1 and 1, respectively. Content validity was confirmed by a panel of 10 experts in the fields of health education, public health, and nutrition. To examine the reliability of the HBM questionnaire by the Cronbach’s alpha test, it was tested among the 20 mothers referring to public health centers with similar demographic characteristics as our study participants, and the result was found to be satisfactory with a Cronbach’s alpha of higher than 0.78. The Cronbach’s alpha coefficients for each construct were as follows: Knowledge = 0.81, Perceived Benefits = 0.88, Perceived Barriers = 0.85, Perceived Susceptibility = 0.62, Perceived Severity = 0.67, Cues to action = 0.53, and Self-efficacy = 0.75. The internal consistency of the questionnaire was determined with a Cronbach’s alpha coefficient of 0.87.

### Assessment of quality-of-life questionnaire

Also, the impact of health on an individual’s everyday life was assessed by the Persian version of the questionnaire (SF-12)^33^. It includes 12 questions and 8 scales including physical functioning, role limitations due to physical problems, bodily pain, general health, vitality, social functioning, role limitations due to emotional problems, and perceived mental health. Response categories for each vary from 2- to 6-point scales and raw scores for items range from 1 to 6. After recording raw scores for some items; then the raw scores could be transformed to provide eight scale scores each ranging from 0 (the worst) to 100 (the best), with higher scores indicating a better quality of life.

### General and physical activity questionnaires

For the initial assessment, we administered a general questionnaire including maternal age, education, income status, number of children, pregnancy intention, job status, and intake of iron and folic acid. Pre-pregnancy BMI and gestational weight gain were recorded from participants’ health records. At the baseline and end of the study, a short version of the International Physical Activity Questionnaire (IPAQ) was utilized to report physical activity as the metabolic equivalent of task (MET) minutes per week^34^.

### Dietary assessment

Participants were instructed to maintain a 3-day food record both at the baseline and end of the study and submit it to the researchers upon completion. A nutritionist verified the accuracy of the records. Dietary intakes were analyzed using Nutritionist 4 (First Data Bank), with national food composition tables used as a reference^35^.

### Biochemical assessment

At the beginning and end of the study, 10 cc of blood was taken from the participants after 8–10 h of fasting to measure the concentration of biochemical factors. Hence, serum was isolated and stored at -80 °C until laboratory analysis. Serum glucose levels were measured during the Oral Glucose Tolerance Test (OGTT) at baseline (fasting), and then 1 h and 2 h after consuming 75 g of glucose. Additionally, concentrations of triglycerides, total cholesterol, low-density lipoprotein (LDL) cholesterol, and high-density lipoprotein (HDL) cholesterol were assessed using a photometric assay (Pars Azmoun). Fasting insulin level was measured using an enzyme-linked immunosorbent assay (MonobindInc). For insulin resistance evaluation, we used the homeostatic model assessment (HOMA-IR) and HOMA IS. Also, hs-CRP concentration was assessed by Elisa kits.

### Statistical analysis

Data were reported as mean and standard deviation (for quantitative data) and frequency (for qualitative data). The normality of the data was checked by Kolmogorov– Smirnov test. We used the paired sample t-test for within groups and the independent sample t-test for between-group comparison. Furthermore, analysis of covariance was used to recognize any differences between the intervention and control groups at the end of the study, adjusting for baseline values and covariates include of; biochemical level at baseline, macronutrients (%) and energy intake, maternal age, pre-BMI, 2nd-trimester weight gain, income status, maternal education level, pregnancy intention. In addition, a chi-square test was applied for qualitative variables. A P-value less than 0.05 was considered significant in all analyses. All of the analyses were performed by SPSS software (version 18; SPSS Inc., Chicago, IL).

## Results

### Patient baseline characteristics and physical activity between groups

A total of 90 pregnant women with gestational diabetes mellitus were enrolled in this study, of these, 6 people were excluded from the study for reasons such as immigration, unwillingness to continue participating in the study, abortion, and incomplete pregnancy monitoring record. Finally, 84 participants were included in the final analysis. The CONSORT flow chart of the study is shown in Fig. 1. As shown in Table 1, The mean age of the participants in the intervention group was 29.97 ± 5.86 years, and in the control, the group was 30.84 ± 5.67 years, which wasn’t significantly different (*P* = 0.4). Maternal pre-pregnancy BMI in the intervention group was 26.40 ± 4.67 kg/m^2^ and in the control group was 27.93 ± 5.34, which was non-significant (*P* = 0.1). As shown in Table 1, at the baseline of the study, there weren’t any significant differences between the two groups in terms of other socio-demographic factors including family income, living with the old person, maternal employment status, pregnancy intention, spouse education status, iron supplement intake, systolic and diastolic blood pressure (*P* > 0.05). However, in terms of number of children, women in the intervention group had significantly more than one child(*P* = 0.004).

In terms of physical activity, at baseline, patients in the intervention group were significantly more active than the control group (979 ± 1022.1 min/week vs. 508.5 ± 837.1 min/week; *P* = 0.01). However, at the end of the study, we didn’t find any significant differences between the two groups in terms of physical activity (*P* = 0.1).

### Comparison of dietary intake between groups

The results for dietary intake are shown in Table 2.

**Table 2:** Maternal intake of energy, macronutrients, and micronutrients during pregnancy between and within study groups.

Variables	Intervention *N* = 42	Control *N* = 42	*P*^3^-value between groups	*P*^4−^value adjusted
Energy (kcal/d)				
Baseline	1657.17(419.33)	1709(445.77)	*P* = 0.5	0.03
Follow-up	1727.39(433.72)	1897.52(445.44)	*P* = 0.07	
P-value^1^	0.4	0.03		
Mean difference	70.22(600.23)	187.99(556.98)		
P- value ^2^	0.4	0.03		
Protein (%)				
Baseline	14.70(4.79)	17.03(4.98)	*P* = 0.02	0.5
Follow-up	14.31(5.01)	14.87(3.96)	*P* = 0.5	
P-value	0.7	0.02		
Mean difference	-0.38(7.26)	-2.15(6.29)		
P- value ^2^	0.7	0.02		
Fat(%)				
Baseline	32.14(8.50)	34.74(7.70)	*P* = 0.1	0.5
Follow-up	29.31(9.60)	31.71(9.19)	*P* = 0.2	
P-value	0.09	0.07		
Mean difference	-2.82(11.06)	-3.03(11.16)		
P- value ^2^	0.09	0.07		
CHO(%)				
Baseline	54.76(11.15)	49.14(9.60)	0.01	0.7
Follow-up	58.18(12.23)	53.28(10.24)	0.04	
P-value	0.1	0.02		
Mean difference	3.42(14.58)	4.13(11.99)		
P- value ^2^	0.1	0.02		
Fiber (g/d)				
Baseline	15.94(8.44)	14.90(8.06)	*P* = 0.5	0.8
Follow-up	20.09(8.94)	18.23(7.35)	*P* = 0.2	
P-value	0.02	0.05		
Mean difference	4.14(12.15)	3.33(11.005)		
P- value ^2^	0.02	0.05		
Potassium				
Baseline	2395.78(1163.21)	2416.73(1066.40)	*P* = 0.9	0.8
Follow-up	2388.35(1039.16)	2242.07(708.48)	*P* = 0.4	
P-value	0.9	0.3		
Mean difference	-7.43(1597.31)	174.65(1252.29)		
P- value ^2^	0.9	0.3		
Magnesium				
Baseline	185.62(96.21)	193.66(102.03)	0.7	0.8
Follow-up	188.27(108.93)	187.06(62.30)	0.9	
P-value	0.9	0.7		
Mean difference	2.64(152.65)	-6.59(120.310		
P- value ^2^	0.9	0.7		
SFA(%)				
Baseline	7.94(3.17)	8.29(3.66)	*P* = 0.6	0.02
Follow-up	6.94(3.43)	8.62(5.39)	*P* = 0.08	
P-value	0.1	0.7		
Mean difference	-0.92(10.53)	3.53(17.89)		
P- value ^2^	0.1	0.7		
β-Carotene				
Baseline	692.69(1391.92)	816.70(1188.16)	*P* = 0.6	0.07
Follow-up	831.29(857.70)	495.16(787.98)	*P* = 0.05	
P-value	0.5	0.1		
Mean difference	138.60(1571.51)	-321.53(1319.48)		
P- value ^2^	0.5	0.1		
Vit E				
Baseline	10.72(11.22)	10.87(10.75)	*P* = 0.9	0.8
Follow-up	12.21(17.92)	18.23(7.35)	*P* = 0.9	
P-value	0.6	0.5		
Mean difference	1.48(21.91)	1.64(17.35)		
P- value ^2^	0.6	0.5		
Vit B6				
Baseline	0.23(0.4)	0.53(0.65)	*P* = 0.01	0.2
Follow-up	0.32(0.54)	0.40(0.49)	*P* = 0.4	
P-value	0.4	0.3		
Mean difference	0.08(0.73)	-0.12(0.84)		
P- value ^2^	0.4	0.3		
Folate				
Baseline	210.67(172.21)	170.73(80.14)	*P* = 0.1	0.5
Follow-up	288.44(201.47)	306.25(174.62)	*P* = 0.6	
P-value	0.05	0.0001		
Mean difference	77.77(263.13)	135.51(196.76)		
P- value ^2^	0.05	0.0001		
Vit B12				
Baseline	2.28(2.61)	2.37(1.71)	*P* = 0.8	0.09
Follow-up	1.82(1.65)	2.36(1.80)	*P* = 0.1	
P^1^-value	0.3	0.9		
Mean difference	-0.46(3.13)	0.008(2.40)		
P- value ^2^	0.3	0.9		
Vit C				
Baseline	98.16(88.54)	84.51(57.21)	*P* = 0.3	0.8
Follow-up	139.60(105.37)	105.30(72.97)	*P* = 0.07	
P-value	0.04	0.1		
Mean difference	41.44(135.56)	20.79(90.73)		
P- value ^2^	0.04	0.1		
Vit K				
Baseline	185.57(277.30)	123.97(97.65)	*P* = 0.1	0.7
Follow-up	289.18(335.70)	271.51(292.33)	*P* = 0.7	
P-value	0.1	0.003		
Mean difference	103.61(448.51)	147.54(313.17)		
P- value ^2^	0.1	0.003		

*Mean (SD).

P-value 1: P values denote the significance of within-group changes (P < 0.05, paired samples t-test or Wilcoxon)P-value 2: P values denote the significance of within-group mean difference (P < 0.05, paired samples t-test)P-value 3: P values denote the significance of between-group difference (P < 0.05, independent samples t-test or U Mann Whitney)P-value 4: P values denote the significance of between-group mean difference (P < 0.05, ANCOVA) in the adjusted model for biochemical baseline levels, macronutrients baseline levels, energy intake baseline levels, maternal age, pre-BMI, 2nd-trimester weight gain, income status, maternal education level, and pregnancy intention.

At baseline participants in the control group compared to the intervention group consumed more protein (17.03 ± 4.98%; 14.70 ± 4.79%, *P* = 0.02) and lower carbohydrate (49.14 ± 9.6%; 54.76 ± 11.15%, *P* = 0.01). While at the end of the study participants in the intervention consumed more fiber (20.09 ± 8.94 *P* = 0.02) than the control group(18.23 ± 7.35 *P* = 0.05). During the study, the intervention group consumed more vitamin C from the beginning (98.16 ± 88.54) to the end of the study (139.60 ± 105.37 *P* = 0.04). The group comparison revealed that the intervention used lower calories than the control group (mean change 70.22 ± 600.23 vs. 187.99 ± 556.98 *P* = 0.03). Additionally consumed lower saturated fat than the control group (mean change − 0.92 ± 10.53 vs. 3.53 ± 17.89 *P* = 0.02). However, we did not observe any additional significant differences between the groups concerning other dietary components.

### Comparison of glycemic indices, lipid profile, and hs-CRP between groups

Table 3 presents a comparison of the mean ± SD of the glycemic indices and lipid profile between groups at the baseline and end of the study. At the baseline of the study, there weren’t any significant differences between the two groups in terms of FBS (*P* = 0.2), insulin (*P* = 0.09), HOMA-IR (*P* = 0.07), and HOMA-IS (*P* = 0.1). After three months of intervention, we found no significant mean differences in glycemic indices between the intervention and control groups (*P* > 0.05). Additionally, the OGTT results at both 1 h and 2 h did not reveal significant differences between the groups (*P* = 0.60 and *P* = 0.80, respectively). However, a significant difference was observed in the adjusted model for hs-CRP levels, with a mean change of -0.877 ± 3.47 in the intervention group compared to -0.067 ± 3.40 in the control group (*P* = 0.01).

**Table 3:** Glycemic indices and lipid profile during pregnancy between and within study groups.

Variables	Intervention *N* = 42	Control *N* = 42	*P*^3^-value between groups	*P*^4−^value adjusted
FBS(mg/dl)				
Baseline	87.46(8.01)	90.09(8.57)	0.1	
Follow-up	88.06(20.22)	85.87(13.35)	0.6	0.29
P- value ^1^	0.8	0.1		
Mean difference	0.593(21.58)	-4.22(15.29)		
P- value ^2^	0.8	0.1		
OGTT1-h(mg/dl)				
Follow-up	133.71(35.62)	134.88(32.79)	0.8	0.9
OGTT2-h(mg/dl)				
Follow-up	97.76(25.07)	101.82(24.98)	0.4	0.8
Fasting insulin (µU/ml )				
Baseline	11.31(4.88)	12.04(5.16)	0.5	0.09
Follow-up	11.29(5.69)	12.52(8.08)	0.4	
P- value ^1^	0.9	0.7		
Mean difference	-0.013(7.15)	0.481(8.74)		
P- value ^2^	0.9	0.7		
hs-Crp(mg/ml)				
Baseline	4.52(2.54)	4.71(2.34)	0.8	0.01
Follow-up	3.64(2.10)	4.65(2.25)	0.04	
P- value ^1^	0.1	0.9		
Mean difference	-0.877(3.47)	-0.067(3.40)		
P- value ^2^	0.1	0.9		
HOMA IS				
Baseline	0.340(0.023)	0.334(0.02)	0.3	0.13
Follow-up	0.345(0.037)	0.339(0.03)	0.2	
P- value ^1^	0.5	0.4		
Mean difference	0.0054(0.044)	0.0048(0.033)		
P- value ^2^	0.5	0.4		
HOMA IR				
Baseline	2.47(1.26)	2.72(1.21)	0.5	0.07
Follow-up	2.58(1.88)	2.73(1.77)	0.6	
P- value ^1^	0.8	0.9		
Mean difference	0.102(2.26)	0.0089(1.87)		
P- value ^2^	0.8	0.9		
TG (mg/dl)				
Baseline	227.58(83.73)	174.87(60.45)	0.0001	0.001
Follow-up	172.34(69.36)	215.80(116.99)	0.04	
P- value ^1^	0.003	0.07		
Mean difference	-55.24(111.21)	40.92(142.01)		
P- value ^2^	0.003	0.07		
LDL(mg/dl)				
Baseline Follow-up	123.19(32.18) 132.96(39.13)	131.99(29.92) 152.94(41.60)	0.1 0.03	0.22
P- value ^1^	0.2	0.01		
Mean difference	9.77(48.36)	20.95(49.64)		
P- value ^2^	0.2	0.01		
HDL(mg/dl)				
Baseline	47.58(11.21)	50.34(6.83)	0.2	0.26
Follow-up	50.90(11.82)	53.82(9.05)	0.2	
P- value ^1^	0.1	0.06		
Mean difference	3.31(13.54)	3.48(11.78)		
P- value ^2^	0.1	0.06		
Total cholesterol(mg/dl)				
Baseline	202.90(42.20)	216.24(38.91)	0.06	0.01
Follow-up	225.65(48.97)	246.36(51.88)	0.6	
P- value ^1^	0.03	0.003		
Mean difference	22.75(66.17)	30.12(61.33)		
P- value ^2^	0.03	0.003		

*Mean (SD).

P-value 1: P values denote the significance of within-group changes (P < 0.05, paired samples t-test or Wilcoxon).P-value 2: P values denote the significance of within-group mean difference (P < 0.05, paired samples t-test).P-value 3: P values denote the significance of between-group difference (P < 0.05, independent samples t-test or U Mann Whitney).P-value 4: P values denote the significance of between-group mean difference (P < 0.05, ANCOVA) in the adjusted model for biochemical baseline levels, macronutrients baseline levels, energy intake baseline levels, maternal age, pre-BMI, 2nd-trimester weight gain, income status, maternal education level, and pregnancy intention.

The baseline of the study showed that participants in the intervention group started with higher levels of TG (227.58 ± 83.73) compared to the control group (174.87 ± 60.45; *P* = 0.001). At the end of the study, the intervention group showed a significant reduction in TG levels compared to the control group in the adjusted model (-55.24 ± 111.21 vs. 40.92 ± 142.01; *P* = 0.001). Additionally, participants in the intervention group had a lower increase in total cholesterol compared to the control group in the adjusted model (22.75 ± 66.17 vs. 30.12 ± 61.33; *P* = 0.01) at the end of the study. However, there were no significant differences between the groups in terms of HDL and LDL concentration (*P* = 0.2 for HDL and *P* = 0.2 for LDL).

### Comparison of health believe model subscales between groups

The results of the patient’s health belief model (HBM) constructs at the beginning and end of the study are shown in Table 4. At the beginning of the study, we didn’t find any significant differences between the two groups in terms of health belief model construct components (*P* > 0.05). In the within-group analysis, at the end of the three-month intervention baseline, we found a significant increase in the perceived susceptibility (mean change: 1.45 ± 4.21; *P* = 0.03) and cue to action (mean change: 1.22 ± 3.38; *P* = 0.02). However, the results of the between-group comparison showed that at the end of the study, there was no significant difference between the two groups in terms of HBM indicators (*P* > 0.05).

**Table 4:** Mean scores of the health belief model constructs during pregnancy between and within study groups.

Variables*	Intervention *N* = 42	Control *N* = 42	*P*^3^-value between groups	*P*^4−^value adjusted
Perceived susceptibility				
Baseline	20.00(3.93)	19.84(4.46)	0.9	0.06
Follow-up	21.45(3.37)	21.58(3.89)	0.9	
P- value ^1^	0.03	0.01		
Mean difference	1.45(4.21)	1.74(4.30)		
P- value ^2^	0.03	0.01		
Perceived severity Baseline Follow-up P- value ^1^ Mean difference P- value ^2^	36.80(5.04) 36.97(5.4) 0.8 0.175(6.49) 0.8	36.51(6.88) 37.87(5.13) 0.1 1.35(5.59) 0.1	0.9 0.5	0.9
Perceived benefits Baseline Follow-up P-value ^1^ Mean difference P-value ^2^	26.73(5.04) 27.65(4.10) 0.3 0.92(6.19) 0.3	27.80(4.35) 28.85(4.91) 0.2 1.04(5.41) 0.2	0.1 0.2	0.8
Perceived barriers Baseline Follow-up P-value ^1^ Mean difference P-value ^2^	27.4(7.32) 28.47(6.43) 0.4 1.07(8.08) 0.4	28.41(7.53) 30.19(7.54) 0.06 1.78(5.95) 0.06	0.5 0.3	0.4
Self-efficacy Baseline Follow-up P-value ^1^ Mean difference P-value ^2^	27.97(3.75) 28.70(3.62) 0.3 0.731(4.66) 0.3	28.05(4.00) 27.5(4.87) 0.5 -0.55(5.11) 0.5	0.6 0.1	0.09
Cue to action Baseline Follow-up P-value ^1^ Mean difference P-value ^2^	19.2(3.32) 20.42(2.92) 0.02 1.22(3.38) 0.02	19.56(3.32) 20.65(3.94) 0.1 1.09(4.64) 0.1	0.4 0.6	0.5
Knowledge Baseline Follow-up P-value ^1^ Mean difference P-value ^2^	34.07(4.72) 35.00(4.42) 0.2 0.921(5.30) 0.2	35.02(4.90) 35.00(5.57) 0.9 -0.02(4.69) 0.9	0.3 0.8	0.1

*Mean (SD).

P-value 1: P values denote the significance of within-group changes (P < 0.05, paired-samples t-test or Wilcoxon).P-value 2: P values denote the significance of within-group mean difference (P < 0.05, paired samples t-test).P-value 3: P values denote the significance of between-group differences (P < 0.05, independent samples t-test or U Mann Whitney).P-value4: P values denote the significance of between-group mean difference (P < 0.05, ANCOVA) in the adjusted model for biochemical baseline levels, macronutrients, energy intake, maternal age, pre-BMI, 2nd-trimester weight gain, income status, maternal education level, pregnancy intention.

The results of the SF-12 domains short-form health survey are shown in Table 5. As shown, at baseline there weren’t any significant differences between the two groups in terms of SF-12 domains short-form health survey (*P* > 0.05). After three months of intervention, the subscale of general health was improved in the intervention compared to the control group in the adjusted model (mean change 13.69 ± 29.83 vs. 0.00 ± 29.58; *P* = 0.04). However, in other subscales, we did not find any significant differences between the two groups or in each group at the end of the study (*P* > 0.05).

**Table 5:** Baseline and follow-up scores for the SF-12 domains short-form health survey during pregnancy between and within study groups.

Variables	Intervention *N* = 42	Control *N* = 42	*P*^3^-value between groups	*P*^4−^value adjusted
Physical function Baseline Follow-up P- value ^1^ Mean difference P-value ^2^	48.21(36.78) 50.59(35.56) 0.7 2.38(47.75) 0.7	58.53(33.82) 54.87(36.75) 0.6 -3.65(44.56) 0.6	0.07 0.5	0.1
Role physical Baseline Follow-up P- value ^1^ Mean difference P-value ^2^	45.53(30.22) 47.91(27.59) 0.6 2.38(38.07) 0.6	55.79(31.51) 53.35(28.22) 0.6 -2.43(37.10) 0.6	0.06 0.3	0.2
Bodily pain Baseline Follow-up P-value ^1^ Mean difference P-value ^2^	74.40(28.99) 67.26(31.47) 0.1 -7.14(31.85) 0.1	71.34(20.58) 65.85(27.24) 0.2 -5.48(30.87) 0.2	0.7 0.8	0.8
General health Baseline Follow-up P-value ^1^ Mean difference P-value ^2^	48.21(20.94) 61.90(24.83) 0.005 13.69(29.83) 0.005	53.04(21.79) 53.04(23.18) > 0.5 0.00(29.58) > 0.5	0.4 0.09	0.04
Social function Baseline Follow-up P-value ^1^ Mean difference P-value ^2^	58.33(30.07) 52.38(35.70) 0.4 -4.76(43.20) 0.4	65.24(34.40) 64.02(31.14) 0.8 -1.21(41.43) 0.8	> 0.5 0.6	0.2
Role emotional Baseline Follow-up P-value ^1^ Mean difference P-value ^2^	58.92(29.51) 64.88(25.63) 0.2 5.95(31.98) 0.2	66.15(31.02) 65.54(29.01) 0.9 -0.60(42.84) 0.9	0.2 0.9	0.3
Mental health Baseline Follow-up P-value ^1^ Mean difference P-value ^2^	70.53(22.22) 65.17(20.20) 0.1 -5.35(22.45) 0.1	66.46(22.43) 67.07(24.16) 0.8 0.60(29.70) 0.8	0.3 0.6	0.2
Vitality Baseline Follow-up P-value ^1^ Mean difference P-value ^2^	58.33(30.07) 52.38(35.70) 0.3 -5.95(42.70) 0.3	62.19(30.68) 59.14(29.45) 0.6 -3.04(37.99) 0.6	0.5 0.3	0.3
Mental component score(MCS) Baseline Follow-up P-value ^1^ Mean difference P-value ^2^	47.66(10.26) 46.37(9.73) 0.4 -1.29(11.89) 0.4	47.25(11.56) 47.47(11.48) 0.9 0.22(15.57) 0.9	0.7 0.6	0.2
Physical component score(PCS) Baseline Follow-up P-value ^1^ Mean difference P-value ^2^	39.54(10.81) 40.76(8.85) 0.5 1.21(12.25) 0.5	42.36(9.81) 40.76(10.01) 0.4 -1.60(12.24) 0.4	0.1 0.5	0.09

*Mean (SD).

P-value 1: P values denote the significance of within-group changes (P < 0.05, paired-samples t-test or Wilcoxon).P-value 2: P values denote the significance of within-group mean difference (P < 0.05, paired samples t-test).P-value 3: P values denote the significance of between-group differences (P < 0.05, independent samples t-test or U Mann Whitney).P-value4: P values denote the significance of between-group mean difference (P < 0.05, ANCOVA) in the adjusted model for biochemical baseline levels, macronutrients, energy intake, maternal age, pre-BMI, 2nd-trimester weight gain, income status, maternal education level, pregnancy intention.

## Discussion

The findings of the present study showed that the application of the HBM approach in nutritional education can have positive effects on some aspects of patients’ beliefs and nutritional behavior. Additionally, pregnant women in the HBM approach demonstrated a significant reduction in triglyceride (TG) concentrations compared to the control group. At the end of the study, the increase in total cholesterol (TC) concentrations among the intervention group was also less pronounced than that of the control group, and this difference was statistically significant. Furthermore, between-group comparisons indicated a significantly greater decrease in hs-CRP levels in the intervention group than in the control group.

This study showed that there was no significant difference in the perceived susceptibility and cues to action scores between the intervention and control groups before the intervention, but there was a significant increase after the educational intervention in the intervention group.

This study found no significant differences in perceived susceptibility and cues to action scores between the intervention and control groups before the intervention. However, following the educational intervention, there was a significant increase in these scores within the intervention group.

In line with our findings, Mohebbi et al. in a Quasi-experimental study found that self-management education led to a significant improvement in perceived susceptibility^36^.

At the beginning of this study, we found that the women in the intervention group compared to the control group consumed more carbohydrates and lower protein. However, after the intervention period, the intervention group significantly increased fiber intake. Diets with low glycemic index emphasize vegetables, legumes, and whole grains as good sources of fiber. In fact, in the present study, after the intervention, the percentage of carbohydrates consumed in the intervention group increased, but it seems that this increase was high in complex carbohydrates and high fiber. Sasanfar et al. in an interventional study among 227 women evaluated the effects of nutritional education sessions based on the HBM approach. They found that after intervention time, women reduced carbohydrate and protein consumption and increased their intake of whole grains, low-fat dairy, and nuts^37^. Recently, an interventional study by Dehghan T et al. showed a positive link between different nutritional education approaches and overall dietary acquaintance in individuals with type 2 diabetes^38^. Likewise, Staynova et al. found out that a printed educational booklet on awareness of the disease in women with gestational diabetes mellitus can improve patients’ understanding of management, as well as their health literacy and motivation^39^. A previous cross-sectional study conducted by Gastrich MD et al. assessed the knowledge and beliefs of pregnant women about gestational diabetes mellitus awareness during prenatal care. The study recommended that targeted education is essential to help women gain a better understanding and reduce their risk of gestational diabetes mellitus^40^ .

Biological and cultural influences such as taste, sex, and age may have significant effects on food consumption. This means that rich people do not always have better food choices, and education plays a vital role in this context^41^. In our study, although there was no statistically significant difference in the HBM subscale, all of these items had a positive trend at the end of the study compared to the beginning. Hence, it is important to develop an educational approach related to maternal’ perception of healthy behavior to improve gestational health status.

The HBM would appear to be utilized broadly for communication investigation^42^. In our study, the score of cues to action increased after the intervention. This implies that the opinion of women on the accessibility of high-fiber cereal and vegetables for the prevention of gestational diabetes mellitus increased. Similarly, to our findings, an interventional study among 84 patients with gastric cancer, found that nutrition education programs based on the HBM model led to a significant improvement in the total score^43^.

The results of the present study indicated that, while most subscales related to the SF-12 showed improvements after the intervention, only the increase in the general health subscale was statistically significant when comparing the intervention group to the control group.

Various studies have evaluated the effect of low glycemic index diets on common psychological disorders. Haghighatdoost et al. in a meta-analysis study evaluated the association between glycemic index, glycemic load, and common psychological disorders. They found that higher GI was associated with lower quality of life and increased risk of depression^44^.

In addition, we found that nutrition education of GI based on the HBM model led to a significant reduction in TG concentration and a lower increase in TC and LDL. However, in contrast to the obvious benefit of a low-GI diet concerning its effect on glycemic status, we did not find significant differences in glycemic indices. Contrary to our findings, Mohebbi et al. found that significant reduction in HbA1c concentration^36^. Rizkalla et al. in a randomized clinical trial evaluated the effects of a low glycemic diet on plasma glucose and lipid profile among patients with type 2 diabetes and found that a four-week low glycemic index diet led to a significant reduction in fasting blood glucose, TC, and LDL^45^. Similarly, in a study by Lv et al., 134 women with gestational diabetes mellitus were assigned to either conventional nutrition or nutritional intervention based on GL. They reported significant differences in fasting blood glucose and the 2 h postprandial glucose levels between the two groups with lower levels in the group receiving intervention^46^.

Because diets with a lower glycemic index contain lower amounts of simple sugars, and saturated fatty acids and increase the consumption of vegetables and fruits^47^.

Additionally, Pooled analysis of low-GI diets showed a more significant decrease in fasting glucose compared with control diets^48–50^.

The finding of a Meta-analysis with the inclusion of 29 trials with moderately controlled diabetes conditions, observed that Low GI/GL dietary patterns reduced HbA1c, fasting glucose, lipids, body weight, BMI, systolic blood pressure (dose-response), and C-reactive protein (CRP) in comparison with higher GI/GL control diets^51^ as pregnancy is a condition that contributed to the rise in inflammatory condition. CRP is a highly detectable protein during the acute phases of diseases and assumed that CRP plays a role in gestational diabetes mellitus^52^ .

Pregnancy is a hyperglycemic period of life and is associated with increasing insulin resistance starting in the second half of the pregnancy period^53^. Therefore, the severity of insulin resistance leads to gestational diabetes mellitus. Moreover, there is an association between abnormal high-sensitivity C-reactive protein (hs-CRP) and pregnancy-specific complications^54^. In the present study among high-risk mothers, hs-CRP was reduced significantly more in the intervention group than control at the end of the study. In line with our finding, Kumari et al. in the case-control study showed an increased level of hs-CRP (76% vs28%) in gestational diabetes mellitus as compared to normal pregnant subjects. Importantly, hs-CRP can be used as a screening tool for early detection and risk assessment of gestational diabetes mellitus^55^ .

### Limitation and strengths

This study had its strengths and limitations. Based on our knowledge, we administered the first clinical trial early in gestation which evaluated the effects of food glycemic index and load education by the HBM model in the Khuzestan province, southwest of Iran. Early pregnancy intervention is essential for favorable modification of maternal weight gain and glycemic status, thereby better preventing the incidence of gestational diabetes mellitus. Moreover, we measured some glycemic indices, lipids, and inflammatory factor hs-CRP along with an assessment of maternal quality of life. However, this study had some limitations. At first, it seems that if the duration of the intervention was 6 months with the inclusion of pregnancy outcomes, more accurate results would have been obtained that may reduce the generalization of the study to the target population. The trial was limited to primary health centers in urban settings, so, may not be broadly generalizable to other rural health centers. Also, some confounding variables such as personality characteristics, mental health, and media might have affected the outcome, which was not assessed. Given that the present study was conducted during the COVID-19 pandemic, caused social isolation and a reduction in physical activity. Accordingly, despite our advice to prevent lifestyle changes during the intervention, decreased opportunities to have adequate physical activity and dietary intake can affect the results. A recent study reported that 47% of the woman met the physical activity guidelines pre-COVID-19 during their pregnancy, this reduced to 23% during the COVID-19 pandemic in the gestational period^56^.

## Conclusion

In conclusion, Lifestyle modification is the forefront prevention for improving maternal health status during pregnancy. The results of the present study showed that nutritional education on the glycemic index and load of food based on HBM had a positive impact on some of the biomarkers and quality of life among pregnant women. However, further studies are needed to confirm the results of the present study and to determine the effect of food glycemic index and load on maternal pregnancy outcomes.

## Electronic supplementary material

Below is the link to the electronic supplementary material.

Supplementary Material 1

Supplementary Material 2

## Data Availability

The datasets used and/or analyzed during the current study are available from the corresponding author on request.

## Abbreviations

FBS: Fasting blood glucose
HOMA-IR: Homeostasis Model of Assessment and Insulin Resistance
hs-CRP: Hypersensitive C reactive protein
HBM: Health Belief Model
GDM: Gestational diabetes mellitus
BMI: Body mass index
GI: Glycemic index
LGI: Low- glycemic index
CVI: Content validity index
CVR: Content validity ratio
HDL-C: High-density lipoprotein cholesterol
LDL: Low-density lipoprotein
TG: Triglyceride
TC: Total cholesterol
HbA1c: Hemoglobin A1C
OGTT: Oral Glucose Tolerance Test
