# Supplementary material for: Nutritional education on health beliefs, metabolic profiles, and quality of life among high-risk pregnant women for gestational diabetes mellitus: a randomized controlled trial
Source: Sci Rep. 2024 Nov 12;14:27712. doi: 10.1038/s41598-024-78447-7 (PMC11558005; doi:10.1038/s41598-024-78447-7)
Supplement: Supplementary file 2 — Supplementary Material 2 [file 41598_2024_78447_MOESM2_ESM.docx]

| **For all 53 items, participants rated their responses using a Likert scale ranging from strongly agree to strongly disagree.** |  |
| --- | --- |
|  |  |
| I may have risk factors for gestational diabetes, but apparently, I have no symptoms. | 1 |
| Gestational diabetes is more common in those who consume high amounts of starches and simple sugars, like sweets and ice cream, than in those who do not take this much. | 2 |
| I believe that pregnancy increases women's risk of developing diabetes. | 3 |
| .  all pregnant women are susceptible to developing gestational diabetes. | 4 |
| believe that our health and illness are ultimately in God's hands, and there is little we can do to change that | 5 |
| Failing to monitor weight gain and blood sugar levels increases the risk of developing gestational diabetes. | 6 |
| I’m concerned that I may struggle to maintain a healthy diet that includes whole grains, fruits, vegetables, legumes, and low-fat dairy products. | 7 |
| I believe that elevated blood sugar levels during pregnancy can pose risks to both the health of the mother and the fetus. | 8 |
| A diet high in starchy foods and simple sugars, such as sweets, ice cream, and jam, raises the risk of neural tube defects. | 9 |
| Consuming a diet high in starchy foods and simple sugars, such as sweets, ice cream, and jam…., can increase the risk of developing gestational diabetes | 10 |
| Having a diet high in starches, fast food, and simple sugars, such as sweets, ice cream, and jam, can lead to excessive weight gain during pregnancy. | 11 |
| If I develop gestational diabetes, there is a chance that my child may be born with a higher birth weight. | 12 |
| If I experience gestational diabetes, there is a risk that my fetus could be stillborn. | 13 |
| .  If I develop gestational diabetes, it could potentially increase the risk of miscarriage. | 14 |
| If I develop gestational diabetes, I might face difficulty during delivery. | 15 |
| Developing gestational diabetes may increase the likelihood of delivering the baby through a cesarean section | 16 |
| Because of the high costs, I am unable to afford food items. | 17 |
| I don’t know the appropriate cooking methods to reduce sugar content in starchy ingredients effectively | 18 |
| We don't have a fruit and vegetable store near our home. | 19 |
| There is no public transportation available near our home. | 20 |
| I don't have convenient access to fiber-rich foods, such as whole grains, fresh fruits, and vegetables. | 21 |
| I lack adequate information about pregnancy care, including weight monitoring, proper nutrition, and taking supplements. | 22 |
| Because of the high cost of fruits, I can't regularly include them in my diet | 23 |
| I can't eat vegetables regularly because I don't have time to clean and wash them. | 24 |
| .  The healthcare professional does not provide sufficient information for preventing diabetes. | 25 |
| A diet rich in whole grains, vegetables and fruits, legumes, nuts, and low-fat dairy products enhances the health of a pregnant woman. | 26 |
| A diet that includes whole grain bread and cereals, vegetables and fruits, legumes, and low-fat dairy products promotes the health of the developing fetus. | 27 |
| Eating whole grains, vegetables, fruits, legumes, and low-fat dairy reduces the risk of neural tube defects in my baby. | 28 |
| A diet that includes whole grain bread and cereals, vegetables and fruits, legumes, nuts, and low-fat dairy products lowers the risk of gestational diabetes. | 29 |
| A diet rich in whole-grain bread and cereals, vegetables and fruits, legumes, and low-fat dairy products helps improve blood sugar levels during pregnancy. | 30 |
| A diet that includes whole grain bread and cereals, vegetables and fruits, legumes, and low-fat dairy products can aid in weight management during pregnancy. | 31 |
| Eating whole vegetables and fruits is effective in managing gestational diabetes. | 32 |
| Seeing or hearing about gestational diabetes and its complications may lead to a dietary preference for foods such as bread and whole grains, vegetables and fruits, legumes, and low-fat dairy products. | 33 |
| The fear of developing gestational diabetes motivates adherence to a proper diet and regular mobility. | 34 |
| My family and husband support me in maintaining a proper diet and getting enough physical activity. | 35 |
| The fear of harming my fetus drives me to maintain a proper diet and physical mobility. | 36 |
| My sources of information for preventing gestational diabetes include radio, television, parents, friends, the health team, my doctor, and midwives. | 37 |
| If I choose to, I can access accurate nutritional information regarding gestational diabetes and its prevention. | 38 |
| Even though prenatal tests are expensive, I can undergo them for the sake of my health. | 39 |
| Although I enjoy white bread and rice, I can reduce their consumption and substitute them with whole-grain bread and products. | 40 |
| I can reduce the consumption of simple sugars, white bread and rice, baked potatoes, soft drinks, juices, cakes, sweets, pre-prepared foods, fried sausages, fast food, breakfast chocolate, and jam. | 41 |
| I can control my blood sugar by eating healthy food | 42 |
| I can follow the recommended diet to prevent gestational diabetes | 43 |
| Even if the people around me don't help me, I can follow the recommended diet to prevent gestational diabetes | 44 |
| Cooking methods can affect blood sugar levels | 45 |
| Skipping meals can affect blood sugar levels | 46 |
| Eating regular meals and snacks plays a role in blood sugar control | 47 |
| The cooking time and method of food preparation can affect the resulting sugar content. | 48 |
| Daily intake of starchy materials (bread and cereals) provides a large amount of daily calories | 49 |
| Using fresh fruits and vegetables in the snack is effective in controlling blood sugar | 50 |
| In the main meals, using boiled and steamed foods is effective in controlling blood sugar | 51 |
| For diabetes prevention and treatment, eat less starchy foods like bread, rice, and potatoes, more vegetables, and adequate fruits, and eggs, low-fat meat, and limit liver, offal, and sugar. | 52 |
| Blood sugar increases faster after consuming sweets and simple sugars | 53 |

**Supplement Table 1** the health belief model questionnaire**.**
